# Prevalence of dementia in the People’s Republic of China from 1985 to 2015: a systematic review and meta-regression analysis

**DOI:** 10.1186/s12889-019-6840-z

**Published:** 2019-05-15

**Authors:** Yangjun Zhu, Hao Liu, Xi-Li Lu, Bo Zhang, Wanwen Weng, Jun Yang, Jun Zhang, Meng-Jie Dong

**Affiliations:** 1 Department of Ultrasound, the First Affiliated Hospital, College of Medicine, Zhejiang University, Hangzhou, 310003 People’s Republic of China; 2 The Department of Nuclear Medicine, the First Affiliated Hospital, College of Medicine, Zhejiang University, 79 QingChun Road, Hangzhou, 310003 People’s Republic of China; 3 Key Laboratory of Precision Diagnosis and Treatment for Hepatobiliary and Pancreatic Tumours of Zhejiang Province, Hangzhou, 310003 People’s Republic of China

**Keywords:** Dementia, Alzheimer’s disease, Vascular dementia, China, Prevalence

## Abstract

**Background:**

In China, the most populous developing country in the world, dementia represents a serious challenge. We performed a large-scale systematic review and meta-regression analysis to elucidate the prevalence of dementia and its subtypes and to identify potential factors underlying the differences between articles.

**Methods:**

A comprehensive literature search was conducted in the following databases to identify studies published up to December 2015: Cochrane Library, CBMDISK, Chongqing VIP, CNKI, PubMed and EMBASE. All statistical analyses (including subtype and meta-regression analyses) were performed with R version 3.3.3.

**Results:**

In total, 51 surveys were selected. The pooled prevalence rates of dementia and its main subtypes, namely, Alzheimer’s disease (AD) and vascular dementia (VAD), for the population aged 55 years and older were 4.03, 2.44 and 1.09%, respectively. The outcomes showed that the meta-regression analysis was affected by the publication year, sample size, region and diagnostic criteria.

**Conclusions:**

Our analysis provided reliable estimates of the prevalence of dementia/ AD/ VD over the past 30 years, which may be affected by education level, and diagnostic criteria. The prevalence of AD/VAD was higher in northern than in southern China, which warrants further study.

**Electronic supplementary material:**

The online version of this article (10.1186/s12889-019-6840-z) contains supplementary material, which is available to authorized users.

## Background

Dementia is a neurological condition that mostly occurs in individuals older than 60 years and has become a significant global problem in most developed and developing countries due to population ageing. Dementia can diminish a patient’s quality of life and lead to high healthcare costs. A recent meta-analysis published by Fiest et al. [1] reported that the pooled prevalence of dementia worldwide among the elderly aged 60 years and older was 4.86% (95% CI: 4.20–5.63%). In China, the most populous developing country in the world, dementia represents a serious challenge. Dementia in mainland China has been investigated by several groups and seems to be increasing, according to recent reviews [2–4]. Our group previously published a systemic review including 25 studies in 2007 [2], and the review demonstrated that the pooled prevalence of dementia in the elderly population aged 60 years and older was 2.8% (95% CI: 2.5–3.1%). Wu et al. [4] revealed the pooled prevalence of dementia from the 76 studies was 3.8% (95% CI: 3.4, 4.2) with the range from 0.6 to 17.2% for the population aged 60 and over in 2013. Wu et al. [4] was also the first to separate mainland China into three areas (north, central and south) and to discuss the prevalence of dementia in an age-standardized pattern. The authors showed an increasing prevalence from north to south and that the discrepancy between the areas could not be explained by the differences in methodological factors, including the age range and diagnostic criteria. However, Wu et al. [4] did not analyse the prevalence of dementia subtypes (primary Alzheimer’s disease (AD) and vascular disease (VAD)) and did not discuss their findings in the context of chronology. This article provides a detailed study of dementia and its subtypes in mainland China up to December 2015 and attempts to identify potential factors underlying the differences between articles through a meta-regression analysis.

## Methods

### Search strategy and selection criteria

Two reviewers independently searched the Cochrane Library, CBMDISK, Chongqing VIP, CNKI, PubMed and EMBASE databases up to December 2015 using ‘Alzheimer’s disease’ or ‘AD’, ‘vascular dementia’ or ‘VAD’, ‘dementia’, and ‘prevalence’ or ‘associated risk’ or ‘infection status’ or ‘epidemic status’ or surveillance’ as the major search terms. There were no language limitations, but the study did not include epidemiological studies in the areas of Hong Kong, Macao and Taiwan because these data were not easily accessible. In addition, the authors screened the reference lists of the identified articles and corresponded with the study investigators using the approach recommended by the Preferred Reporting Items for Systematic Reviews and Meta-Analyses (PRISMA) guidelines.

The inclusion criteria were as follows: (i) case collection based on a field survey; (ii) studies published in peer-reviewed journals; (iii) the population in the investigations was 55 years and older; (in our study, data from participants below 55 years of age were omitted); and (iv) used a validated method to assess the dementia diagnosis (through both a screening phase and a diagnostic phase). The exclusion criteria were as follows: (i) studies that used the Newcastle-Ottawa scale (NOS); (ii) articles with repeated cases; (iii) articles of inferior quality or with incomplete information; and (iv) unavailable articles (i.e., reviews, comments, and abstracts).

### Data extraction

In the screening stage, all samples were screened using brief cognitive tests, including the Mini-Mental State Examination (MMSE), the Blessed Dementia Scale (BDS) and the Hasegawa Dementia Scale (HDS). Positive screens were identified when the samples scored below the cut-off point on one or more tests or were clinically diagnosed with dementia. In the diagnosis stage, positive screens were further diagnosed by experienced physicians with the assistance of laboratory and neuropsychological tests.

The clinical diagnostic criteria used in the 50 studies included the DSM-III (the Diagnostic and Statistical Manual of Mental Disorders, Third Edition) [5–7], DSM-III-R (the Diagnostic and Statistical Manual of Mental Disorders, Third Edition) [8–23], DSM-IV (the Diagnostic and Statistical Manual of Mental Disorders, Fourth Edition) [24–39], DSM-IV-R (the Diagnostic and Statistical Manual of Mental Disorders, Fourth Edition, revised) [40–47], ICD-10 (international Classification of diseases-10) [5, 13, 15] and CCMD (Chinese Classification and Diagnostic Criteria of Mental Disorders) [48]. The NINCDS-ADRDA (National Institutes of Neurological Disorders and Stroke-Alzheimer’s Disease and Related Disorders Association) criteria were used to diagnose probable AD, and the NINDS-AIREN (National Institute of Neurological Disorders and Stroke and Association International pour la Recherche et 1′Enseignement en Neurosciences) criteria and the Hachinski Ischaemia Score were used to diagnose VAD. Other dementias, such as mixed dementia, dementia with Lewy bodies, Parkinson’s disease with dementia, and alcoholic dementia, were defined using globally accepted criteria.

The detailed information extracted from the articles included the following: (i) basic information, including the author, type of publication and publication year; (ii) study design and tools, such as screening methods and tools, diagnostic criteria and sampling methods; (iii) characteristics of the study population, including age, location, occupation and education; and (iv) the prevalence of dementia and its subtypes.

Full manuscripts of the potentially acceptable studies were screened by two reviewers. The same two authors independently assessed the risk of bias of these nonrandomized studies using a modified version of the Newcastle-Ottawa Scale (NOS), which assesses the quality of nonrandomized studies based on design, content and ease of use to facilitate the task of incorporating quality assessments into the interpretation of meta-analytic results. Disagreements were resolved via consensus after discussion with a third author.

### Statistical analysis and heterogeneity

The prevalence was calculated with the variance-stabilizing double arcsine transformation and weighted towards 50% to include studies with a low dementia prevalence [49]. The 95% confidence intervals (CIs) were calculated using the Wilson method [50]. Two-sided *p* values < 0.05 were considered significant. A *p* value of Cochrane’s Q test < 0.1 or an I^2^ > 50% was defined as indicating significant heterogeneity between studies. Due to the high heterogeneity observed (I^2^ > 75%), random effects models were used for the pooled statistical analyses. To identify factors that influenced the prevalence of dementia and its subtypes (AD and VAD), the characteristics of the cases, including gender, age, occupation, educations, and residence, were divided into diverse groups for analysis. We also performed subgroup analyses based on the specific region or publication year of the study. Publication bias was estimated using Egger’s test.

Due to the high heterogeneity, we further investigated the potential sources of heterogeneity via a meta-regression analysis. All studies were allocated into diverse groups according to their potentially relevant characteristics. The suspected factors were analysed using a univariate model including the (i) publication year (before and including the year 2000 versus after 2000), (ii) geographical region (north China versus central and south China), (iii) living area (urban only versus rural only and mixed), (iv) sample size (> 3000 versus ≤3000), and (v) diagnostic method (DSM−/IV/IV-R versus others). Furthermore, we subdivided the geographical regions/diagnostic methods into 7/4 groups, respectively, and analysed these factors using a multivariate analysis model.

All the analyses were performed with R version 3.3.3 (Microsoft Corporation, Redmond, WA, USA). The PRISMA guidelines were followed throughout the entire process.

### Data availability

All data generated or analysed during this study are included in this published article (and its Supplementary Information files).

## Results

### Study inclusion

**Fig. 1:**
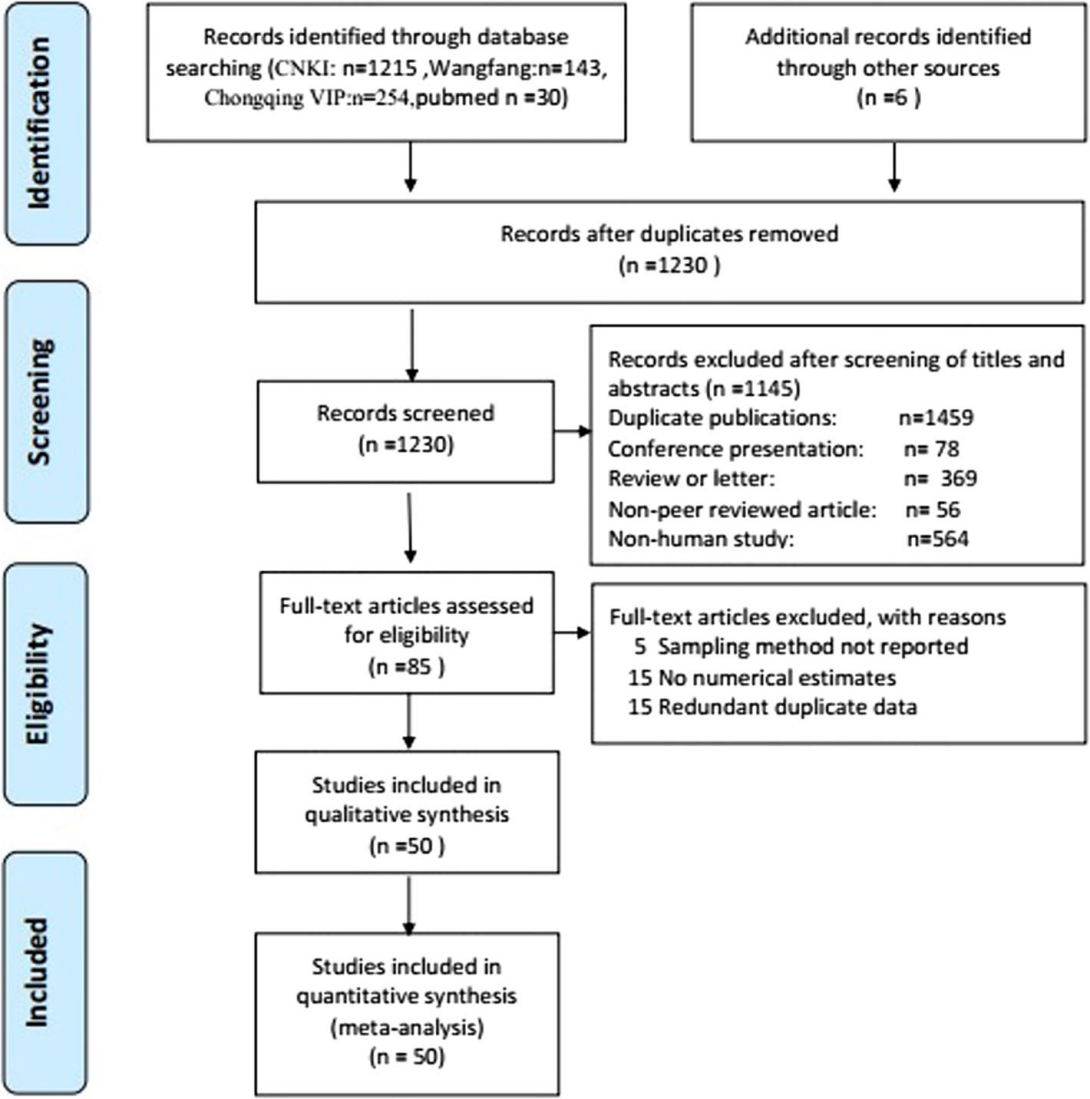
The detailed search process

We strictly screened 1230 studies in a step-by-step manner based on the inclusion criteria. Ultimately, 51 surveys from 50 articles were included after the search process, including 49 studies that reported one survey and 1 study that reported two surveys due to differences in nationality (Han/Kazak nationalities) **(**Fig. 1**)**.

### Descriptive statistics

A total of 166,068 people aged 55 years or older (including 78,603 males and 87,465 females) were gathered from all 51 surveys; the publication year ranged from 1985 to 2015. The locations of all 51 surveys were distributed into 3 areas, including 7 regions (north area: north/northeast/northwest China; central area: central/east China; and south area: south/southwest China) and then subdivided into 23 provinces and municipalities. Detailed information regarding the study characteristics is shown in Table 1.

**Table 1:** Characteristics of the included studies included in this article

study	Location	Urban/rural	Survey date	Subject no.(M/F)	AD	VAD	Dementia	Age range	Diagnostic criteria	Newcastle-Ottawa scale
Chen changhui1992 [9]	Beijing	C	1986.1–1986.6	5172(2385/2787)	10	26	39	≥60	DSM-III	8
gao surong1989 [10]	Beijing	C	1986.4–1986.6	906(409/497)	9	24	35	≥60	DSM-III	8
gao zhixu 1993 [11]	Shanghai	C + R	1990.10–1990.12	3779(1499/2280)	119	32	159	≥60	DSM-III-R	9
wang dao1996 [12]	Shanghai	C + R	1991	1515(610/905)	27	4	33	≥55	DSM-III-R	8
mao ruihe1993 [13]	Fujian	C + R	1992.6–1992.9	1982(787/1195)	29	22	58	≥60	DSM-III-R	9
xue guanhua1997 [7]	Guangdong	C + R	1994	3285(1579/1706)	23	18	47	≥60	DSM-III-R	9
tang mouni1999 [14]	Sichuan	R	1994	5987(2653/3334)	86	11	104	≥65	DSM-III-R	8
li zengjin1997 [15]	Beijing	C	1994.11–1995.5	1027(372/655)	38	25	63	≥60	DSM-III-R	8
chen zhanying1998 [16]	Xinjiang	C + R	1995.1–1995.3	2687(1380/1307)	11	37	48	≥55	DSM-III	8
lv shuchen1998 [51]	Zhejiang	C	1995.10–1996.6	1689(823/866)	101	17	119	≥60	DSM-III-R	8
zhang jingli1998 [25]	Beijing	C	1995.12–1996.12	1243(760/483)	17	12	29	≥60	DSM-III-R	7
tang zhe2002 [52]	Beijing	C + R	1996–1998	2788(1356/1432)	140	43	208	≥60	DSM-III-R	9
wang tianxiang1999 [17]	Anhui	C + R	1997	2749(998/1751)	109	27	143	≥65	ICD-10	9
zhou fen2001 [26]	Shanghai	C + R	1997	15,910(7148/8762)	344	98	478	≥55	DSM-IV	9
lishuran1999 [27]	Beijing	C	1997	1593(697/896)	22	15	40	≥60	ICD-10	7
tang mouni2001 [18]	Sichuan	C + R	1997.6–1998.4	5353(2552/2801)	110	20	143	≥55	DSM-III-R	9
xiao zhijie1999 [19]	Hunan	C + R	1997.6–1998.5	3287(1551/1736)	47	26	86	≥55	DSM-IV	9
qu qiumin2001 [28]	Shanxi	C + R	1997.9–1998.12	4850(2040/2810)	100	54	172	≥55	DSM-IV	9
zhang zhanxing [20]	Shanghai	C + R	1999	1186(597/589)	11	5	33	≥55	DSM-III-R	9
fan jianxiong2000 [48]	Jiangsu	C	1999.5–1999.6	3268(1589/1679)	31	16	48	≥60	DSM-III-R	9
ma cui2005 [40]	Guangdong	C + R	2000	3780(1539/2241)	128	44	182	≥65	DSM-IV	9
tang mouni2005 [29]	Sichuan	C + R	2000.10–2001.3	3908(1924/1984)	78	18	107	≥55	DSM-III-R	9
gongjianbing2002 [53]	Hainan	C + R	2001.10–2001.12	2961(1210/1751)	25	9	39	≥60	CCMD-III-R	9
zhou kaili2002 [21]	Chongqing	C	2001.3–2001.5	1519(611/908)	73	9	87	≥65	DSM-IV-R	9
gao quwen2004 [54]	Guangdong	C	2002	1839(1727/112)	22	40	69	≥60	DSM-IV	7
yuan yefeng2005 [30]	Jiangxi	C + R	2002.3–2002.4	2126(1053/1073)	72	14	93	≥60	ICD-10	8
chen weixiong2004 [31]	Hainan	C	2002.5–2002.10	12,628(6193/6435)	80	113	193	≥60	DSM-III-R	9
li wenbiao2003 [32]	Inner Mongolia	C	2002.7–2002.8	2324(1846/478)	31	31	62	≥60	ICD-10	7
li keqing2008 [33]	Hebei	C + R	2004	2126(1100/1026)	166	52	218	≥65	DSM-IV	9
huang wenyong2007 [22]	Guizhou	C	2005	3229(1227/2002)	41	18	64	≥60	DSM-IV	8
chen bin2009 [55]	Fujian	C	2006.7–2007.4	2373(1039/1334)	81	35	141	≥60	DSM-IV	8
wang hongyan2009 [34]	Shandong	C	2007	618(211/407)	42	10	52	≥60	DSM-IV	7
tan jiehua2007 [35]	Hubei	C + R	2007.1–2007.12	3908(1924/1984)	78	18	107	≥55	DSM-III-R	9
zheng xiuxia2010 [41]	Beijing	C + R	2007.5–2007.9	1756(737/1019)	57	69	126	≥60	ICD-10	9
Zhang honghui 2008 [23]	Fujian	R	2007.7–2007.11	2696(1104/1592)	134	32	166	≥65	DSM-IV	8
fan qinghua2011 [36]	Shanxi	C + R	2008	1826(1126/700)	38	18	74	≥60	DSM-IV	9
wang ying2010 [37]	Liaoning	C	2008.1–2009.1	2100(1136/964)	76	47	143	≥60	DSM-IV-R	7
gao ying2009 [38]	Shanxi	C	2008.3–2008.12	312(163/149)	29	8	37	≥65	DSM-III-R	7
ma yong 2013 [56]	Shanghai	C	2010	2442(1130/1312)	101	89	190	≥65	DSM-IV	8
kang meiyu2011 [42]	Hebei	C + R	2010.1–2010.8	3632(1937/1695)	177	57	263	≥60	DSM-IV	9
meng xinling2014 [39]	Xinjiang	C + R	2010.6–2012.8	2532(1221/1311)	149	68	237	≥55	DSM-IV	9
meng xinling2014 [39]	Xinjiang	C + R	2010.6–2012.8	1078(497/581)	48	21	76	≥55	DSM-IV	9
lao meili2011 [43]	Hainan	C + R	2010.7–2010.8	7665(3509/4156)	111	48	159	≥55	HDS	9
sun hongxian2012 [44]	Shanghai	C	2010–2011	1472(666/806)	56	18	79	≥60	DSM-IV-R	9
cheng qi2013 [57]	Shanghai	R	2011	1472(665/807)	53	21	157	≥60	DSM-IV-R	9
ji yong2015 [58]	Tianjin	R	2011–2012	5578(2482/3096)	299	96	84	≥60	DSM-IV	8
tang jiangping2014 [45]	Hunan	C + R	2011.10–2012.12	10,026(4845/5181)	275	85	395	≥55	DSM-IV	8
wei chongjuan2014 [46]	Tianjin	C	2012	1324(1078/246)	28	28	497	≥55	DSM-IV-R	9
ding ding 2014 [47]	Shanghai	C	2012	3141(1438/1703)	113	25	72	≥60	DSM-IV-R	7
li haihong2015 [59]	Guangxi	C	2012.6–2014.3	889(389/500)	38	18	59	≥60	DSM-IV-R	9
li chonghui2015 [60]	Tianjin	C	2014	2532(1091/1441)	144	59	228	≥60	DSM-IV-R	9

The quality scores of the studies were 7–9 (average = 8.412) when evaluated using the Newcastle-Ottawa quality assessment criteria; the score components for all 50 individual studies are shown in Additional file 1.

### Analysis of outcomes

#### Prevalence of dementia

All 51 surveys provided the number of patients with dementia, which was divided by the different pathological subtypes (AD, VAD and others). Estimates of the dementia prevalence ranged from 0.75–11.86%, and the pooled prevalence of dementia was 4.03% (95% CI: 3.46–4.70%). The pooled prevalence of AD/VAD was 2.44% (95% CI: 2.06–2.89%)/1.09% (95% CI: 0.91–1.31%), with ranges from 0.19–9.29%/0.18–3.64%, respectively (see Additional file 2). For other dementia subtypes, such as Parkinson’s disease, the prevalence (0.09% [0.07%; 0.12%]) was much smaller than that for AD/VAD, as detailed in Additional file 3.

The prevalence of dementia depending on age (55–59, 60–64, 65–69, 70–74, 75–79, 80–84, 85–89, 90–94, and > 95 years; males and females were analysed separately for each age group) is shown in Additional file 4**.** The prevalence of dementia and its subgroups (AD and VAD) increased progressively with increasing age in both males and females.

Other factors, such as sex (male and female), education level (illiterate, primary school, junior high school, senior high school and college), residence (city and rural), and occupation (worker, farmer, officer and housewife) were calculated and presented in Additional file 5**.** The prevalence of AD was significantly higher in females (3.24, 95% CI: 3.08–3.41%) than in males (1.58, 95% CI: 1.46–1.70%), which was not observed for VAD. The prevalence decreased progressively with increasing education level for both AD and VAD. The prevalence rates of AD/VAD were significantly lower in the college group (0.68, 95% CI: 0.31–1.48%)/(0.67, 95% CI: 0.34–1.32%) than in the illiterate group (6.06, 95% CI: 4.08–8.91%)/(2.42, 95% CI: 1.57–3.71%). The prevalence of dementia was not significantly different between the city and rural groups. The housewife group had a much higher prevalence of AD than the other occupations, but the result was biased by gender, because the term “housewife” inherently referred to females.

#### Subgroup analysis

The subgroup analysis based on region showed that the prevalence of AD decreased progressively from north China to south China. The prevalence rates in the northeast/north/northwest areas of north China were 3.62%/3.08%/2.63%, respectively, which were higher than the prevalence rates in the central area (central/east China: 2.25%/3.41%, respectively). The south area (southwest/south China: 2.24%/2.25%, respectively) had the lowest prevalence. The prevalence of VAD in the north region (northeast/north/northwest China: 2.24%/1.62%/1.51%, respectively) was higher than the prevalence in the central and south regions (central/east/southwest/south China: 0.64%/0.99%/0.45%/1.02%, respectively) (see Additional file 6).

We divided the studies into 6 groups according to the publication year (1985–1990, 1991–1995, 1996–2000, 2001–2005, 2006–2010 and 2010–2015) and found that the prevalence rates of AD/VAD were 0.87%/1.04, 1.58%/0.79, 2.09%/0.76, 1.88%/0.94, 3.84%/1.61, and 3.77%/1.49%, respectively (see Additional file 7**)**.

#### Publication bias

**Fig. 2:**
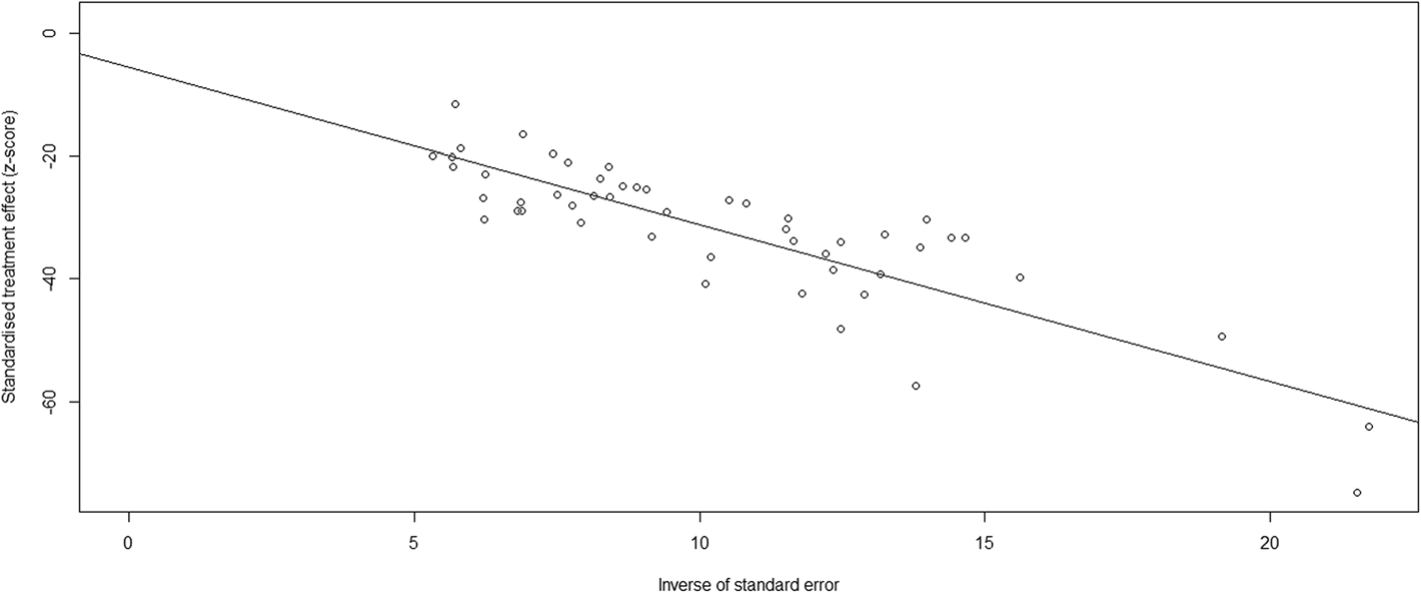
The asymmetric funnel plot from Egger’s test

The *p*-value of Egger’s test was 0.02972. The asymmetric funnel plot showed that publication bias existed between the studies **(**Fig. 2**)**.

#### Meta-regression

The outcomes of the univariate meta-regression analysis are presented in Additional file 8: Table S8 (online)**.** Depending on the univariate meta-regression results, the prevalence rates of dementia/AD/VAD were higher in studies in which the publication year was after 2000 (*p* = 0.0002/0.0002/0.0062), and the sample size was greater than 3000 (*p* < 0.0001/0.0318/0.0025). The prevalence of dementia/AD/VAD was not significantly different according to the living area (neither city versus city plus rural areas nor city versus rural areas) or the diagnostic method (DSM-III/IV/IV-R vs. others). However, the prevalence of VAD was higher in studies from north China (*p* < 0.0001), which was not observed for dementia or AD. Then, we added two risk factors (geographical region and diagnostic criteria) to the multivariate analysis model (Table 2). When we set central China as the reference geographical region, only three groups (north China/northeast China/northwest China) showed higher prevalence rates of VAD (*p* = 0.0006/0.0009/0.0046), which was not observed in dementia or AD. When we set DSM-III/III-R as the reference diagnostic criteria, studies in which DSM-IV/IV-R were used had a higher prevalence of dementia/AD/VAD (p < 0.0001/< 0.0001/0.0008) in the multivariate meta-regression analysis (see Additional file 8**)**.

**Table 2:** Multivariate analysis (random effects model) of the meta-regression

	Meta-regression Coefficient (%)	95% CI	P
Dementia			
Geographical region			
East China	0.2965	−0.4241-1.0171	0.4199
North China	0.3952	−0.3414-1.1319	0.2930
South China	0.0179	−0.7462-0.7820	0.9634
Central China (ref.)	–	–	–
Northeast China	0.7058	−0.3754-1.7871	0.2007
Northwest China	0.2726	−0.6063-1.1514	0.5433
Southwest China	−0.1598	−0.9669- 0.6472	0.6979
Diagnostic criteria			
DSM-III/III-R (ref.)	–	–	–
DSM-IV/IV-R	0.6930	0.3919–0.9940	< 0.0001
ICD-10	0.3147	−0.2421-0.8715	0.2680
HDS	−0.3239	−1.3516-0.7037	0.5367
AD			
Geographical region			
East China	0.4463	−0.4022-1.2948	0.3025
North China	0.3469	−0.5217-1.2155	0.4338
South China	0.0150	−0.8853-0.9154	0.9739
Central China (ref.)	–	–	–
Northeast China	0.5119	−0.7663-1.7900	0.4325
Northwest China	0.1775	−0.8587-1.2137	0.7371
Southwest China	0.0175	−0.9328-0.9679	0.9711
Diagnostic criteria			
DSM-III/III-R (ref.)	–	–	–
DSM-IV/IV-R	0.7478	0.4020–1.0935	< 0.0001
ICD-10	0.2641	−0.3752-0.9034	0.4181
HDS	−0.1414	−1.3096-1.0268	0.8125
VAD			
Geographical region			
East China	0.4028	−0.0984-0.9041	0.1152
North China	0.8777	0.3787–1.3767	0.0006
South China	0.4186	−0.1079-0.9450	0.1192
Central China (ref.)	–	–	–
Northeast China	1.2313	0.5023–1.9603	0.0009
Northwest China	0.8385	0.2546–1.4225	0.0049
Southwest China	0.4186	−0.1079-0.9450	0.1192
Diagnostic criteria			
DSM-III/III-R (ref.)	–	–	–
DSM-IV/IV-R	0.6128	0.2542–0.9713	0.0008
ICD-10	0.6050	−0.0534- 1.2634	0.0717
HDS	−0.2097	−1.4028-0.9835	0.7305

## Discussion

In this systemic review and meta-analysis, we analysed 51 surveys conducted in mainland China from 1985 to 2015. The overall estimate of the dementia prevalence was 4.03% (95% CI: 3.46–4.70%), whereas the pooled prevalence of AD/VAD was 2.44%/1.09%. The prevalence of dementia/AD/VAD was greater than that reported in a previous review [2] but lower than that reported in reviews that selected studies conducted after 2000 [3, 61, 62]; this finding was consistent with the results of a meta-regression that found that the prevalence rates of dementia/AD/VAD were significantly higher in studies published after 2000. The most common type was AD (64.6%), followed by VAD (26.8%). The proportions of AD/VAD were similar to the proportions reported in our previous study [2] but much higher than those observed in United States [63]. Furthermore, in some countries, such as Russia and Japan [64, 65], the prevalence of VAD was higher than that of AD, which might be due to the inclusion of different diagnostic criteria, ethnicities and genetic factors [66, 67].

The prevalence of AD followed an obviously increasing pattern from the southern and central to the northern areas of China in the subgroup analysis. The prevalence of VAD was much higher in the northern area of China, but the difference between the central and southern areas of China was not significant. However, the economic discrepancies and differences in education between these areas were not significant, because all three areas included big cities (Beijing/Shanghai/Guangzhou) and less developed provinces (e.g., Inner Mongolia /Anhui/Guangxi); this finding was consistent with a previous study [4]. Air pollution is much more severe in northern China, and recent studies have shown that PM 2.5 (PM2.5 refers to atmospheric particulate matter (PM) that have a diameter of less than 2.5 μm which is an air pollutant that is a concern for people’s health when levels in air are high) can injure brain structures and cognition, which may ultimately increase the prevalence of AD/VAD. In 2016, an article published by Casanova et al. [68] found that long-term PM 2.5 exposure induced the loss of grey matter and white matter in older women, which could increase the prevalence of AD. Other studies confirmed that air pollution increased the prevalence of dementia [69–71], whereas some larger multi-centre studies published in *Neurotoxicology* and *Lancet* in 2017 also found that the prevalence of dementia was positively related to the level of air pollution [72, 73]. Therefore, we think climate environment factors and other socioeconomic factors may be potential influencing factors that require further research with well-designed studies to identify their impacts.

Chronologically, the prevalence rates of AD/VAD were higher after 2000 than before 2000. Wu et al. [4] showed an increasing trend in the prevalence from 1980 to 2014, but the significance disappeared when the authors accounted for changes in methodological factors (especially the diagnostic criteria). Renewal of the diagnostic criteria for dementia has been ongoing over the past few decades. Several new criteria have been proposed, including DSM–IV/IV–R, which replaced DSM–III/III–R, as well as other older criteria.

The important influence of methodological factors was confirmed by the meta-regression analysis in this study. According to the meta-regression outcomes, studies using the DSM-IV/IV-R had a higher prevalence of dementia/AD/VAD; we suspect that the increased diagnostic level (including the updated diagnostic criteria) partially caused the chronological increases in dementia/AD/VAD.

We found that the prevalence rates of dementia/AD/VAD were negatively correlated with education level [74]. A previous study by Langa et al. [75] reported a significant decline in the prevalence of dementia between 2000 and 2012. The negative relationship between the prevalence of AD and education level has been generally accepted and is consistent with the results of our study. Education was confirmed to be related to the prevalence of AD by former studies based on regional cerebral blood flow [5, 58] and genetic status [64]; thus, education may provide a reserve for the neuropath logical changes of AD, delaying the progress of its clinical manifestations. However, the mechanism underlying the relationship between VAD and education remains unknown and requires further research.

The prevalence of AD was much higher in the female group than in the male group, as previously established. However, the prevalence of VAD did not show a significant difference between the two groups, indicating that gender was not a risk factor for VAD. In the analysis of stratified prevalence based on 5–year age groups, we found a positive relationship between dementia/AD/VAD and age in both the male and female groups. Age was considered the pivotal influencing factor of dementia/AD/VAD. However, as we increased the number of elder groups to include those encompassing ages from 85 to 90 and 90+ years, their total sizes were much smaller than those of the other groups, which might have led to a magnified result. Thus, more high-quality studies are needed to confirm this outcome.

Jia et al. [6] and Ji et al. [8] both reported that the prevalence rates of dementia/AD were higher in rural areas than in urban areas and that education was the most important influencing factor. However, the meta-regression results from this study found no significant difference between urban, rural and mixed areas, which was consistent with the result from Wu et al. [4]. The definition of urban or rural is not simple in China; simply stratifying by location is inaccurate and insufficient. Thus, more studies with stricter designs are needed in the future.

Our study has several limitations. Significant differences in the publication year, sample size and other factors were apparent, although the quality of the studies indicated their reliability. Depending on the results of the multivariate meta-regression analysis, we observed that the high heterogeneity between studies was caused by these risk factors. However, many risk factors were not considered. To obtain more reliable results, these risk factors and other potential factors should be better controlled. More information is needed from studies with identical designs in the future.

Because dementia is a serious societal problem, its prevention deserves more attention. According to the results of our study, some factors, such as air quality and education, are important for the prevention of dementia. However, more high-quality studies are required to resolve the many unknown factors related to dementia.

## Conclusions

The prevalence of dementia/AD/VAD has increased over the past 30 years and may be affected by education level and diagnostic criteria. The prevalence of AD/VAD was higher in northern than in southern China, which warrants further study.

## Additional files

Additional file 1: The quality scores of the studies. (DOC 100 kb)

Additional file 2: The pooled prevalence of AD (A) /VAD (B) /dementia (C). (DOC 829 kb)

Additional file 3: The prevalence of subtypes of dementia included in the studies. (DOC 108 kb)

Additional file 4: The prevalence of dementia in terms of age in China from 1985 to 2015. (DOC 39 kb)

Additional file 5: Prevalence of dementia due to sex, education, occupation and residence difference. (DOC 36 kb)

Additional file 6: The subgroup analysis the prevalence of AD (A) /VAD (B) /dementia (C) based on region. (DOC 543 kb)

Additional file 7: The subgroup analysis the prevalence of AD (A) /VAD (B) /dementia (C) based on published year. (DOC 629 kb)

Additional file 8: Univariate meta-regression of the prevalence of dementia/AD/VAD. (DOC 40 kb)

## Abbreviations

AD: Alzheimer’s disease
BDS: Blessed Dementia Scale
CCMD: Chinese Classification and Diagnostic Criteria of Mental Disorders
CIs: Confidence intervals
DSM-III: The Diagnostic and Statistical Manual of Mental Disorders, Third Edition
DSM-III-R: The Diagnostic and Statistical Manual of Mental Disorders, Third Edition
DSM-IV: The Diagnostic and Statistical Manual of Mental Disorders, Fourth Edition
DSM-IV-R: The Diagnostic and Statistical Manual of Mental Disorders, Fourth Edition, revised
HDS: Hasegawa Dementia Scale
ICD-10: International Classification of diseases-10
MMSE: Mini-Mental State Examination
NINCDS-ADRDA: National Institutes of Neurological Disorders and Stroke-Alzheimer’s Disease and Related Disorders Association
NINDS-AIREN: National Institute of Neurological Disorders and Stroke and Association Internationale pour la Recherche et 1′Enseignement en Neurosciences
NOS: Newcastle-Ottawa scale
PRISMA: Systematic Reviews and Meta-Analyses
VAD: Vascular dementia
