# Supplementary material for: Prevalence of dementia in the People’s Republic of China from 1985 to 2015: a systematic review and meta-regression analysis
Source: BMC Public Health. 2019 May 15;19:578. doi: 10.1186/s12889-019-6840-z (PMC6521412; doi:10.1186/s12889-019-6840-z)
Supplement: Supplementary file 1 — The quality scores of the studies. (DOC 100 kb) [file 12889_2019_6840_MOESM1_ESM.doc]

The quality scores of the studies

| Newcastle-Ottawa scale（NOS） | | | | | | | | |
| --- | --- | --- | --- | --- | --- | --- | --- | --- |
| study | Selection of study population（score four） | | | |  | Outcome（score three） | | |
|  | truly or somewhat representative of the average exposed cohort in the community（score one） | drawn from the same community as the exposed cohort（score one） | structured interview（score one） | Demonstration that outcome of interest was not present at start of study（score one if yes） | study controls for select the most important factor（score one） | ①independent blind assessment*②record linkage（score one） | study location both city and rural area （score two if yes; score one if no） | ①complete follow up - all subjects accounted for* ②subjects lost to follow up unlikely to introduce bias - small number lost（score one） |
| chen changhui1992 | 1 | 1 | 1 | 1 | 1 | 1 | 1 | 1 |
| gao surong1989 | 1 | 1 | 1 | 1 | 1 | 1 | 1 | 1 |
| gao zhixu 1993 | 1 | 1 | 1 | 1 | 1 | 1 | 2 | 1 |
| wang dao1996 | 1 | 1 | 1 | 1 | 1 | 1 | 1 | 1 |
| mao ruihe1993 | 1 | 1 | 1 | 1 | 1 | 1 | 2 | 1 |
| xue guanhua1997 | 1 | 1 | 1 | 1 | 1 | 1 | 2 | 1 |
| tang mouni1999 | 1 | 1 | 1 | 1 | 1 | 1 | 1 | 1 |
| li zengjin1997 | 1 | 1 | 1 | 1 | 1 | 1 | 1 | 1 |
| chen zhanying1998 | 1 | 1 | 1 | 1 | 1 | 1 | 1 | 1 |
| lv shuchen1998 | 1 | 1 | 1 | 1 | 1 | 1 | 1 | 1 |
| zhang jingli1998 | 0 | 1 | 1 | 1 | 1 | 1 | 1 | 1 |
| tang zhe2002 | 1 | 1 | 1 | 1 | 1 | 1 | 2 | 1 |
| wang tianxiang1999 | 1 | 1 | 1 | 1 | 1 | 1 | 2 | 1 |
| zhou fen2001 | 1 | 1 | 1 | 1 | 1 | 1 | 2 | 1 |
| lishuran1999 | 1 | 1 | 1 | 1 | 0 | 1 | 1 | 1 |
| tang mouni2001 | 1 | 1 | 1 | 1 | 1 | 1 | 2 | 1 |
| xiao zhijie1999 | 1 | 1 | 1 | 1 | 1 | 1 | 2 | 1 |
| qu qiumin2001 | 1 | 1 | 1 | 1 | 1 | 1 | 2 | 1 |
| zhang zhanxing2000 | 1 | 1 | 1 | 1 | 1 | 1 | 2 | 1 |
| fan jianxiong2000 | 1 | 1 | 1 | 1 | 1 | 1 | 2 | 1 |
| ma cui2005 | 1 | 1 | 1 | 1 | 1 | 1 | 2 | 1 |
| tang mouni2005 | 1 | 1 | 1 | 1 | 1 | 1 | 2 | 1 |
| gongjianbing2002 | 1 | 1 | 1 | 1 | 1 | 1 | 2 | 1 |
| zhou kaili2002 | 1 | 1 | 1 | 1 | 1 | 1 | 2 | 1 |
| gao quwen2004 | 0 | 1 | 1 | 1 | 1 | 1 | 1 | 1 |
| yuan yefeng2005 | 1 | 1 | 1 | 1 | 1 | 1 | 1 | 1 |
| chen weixiong2004 | 1 | 1 | 1 | 1 | 1 | 1 | 2 | 1 |
| li wenbiao2003 | 0 | 1 | 1 | 1 | 1 | 1 | 1 | 1 |
| li keqing2008 | 1 | 1 | 1 | 1 | 1 | 1 | 2 | 1 |
| huang wenyong2007 | 1 | 1 | 1 | 1 | 1 | 1 | 1 | 1 |
| chen bin2009 | 1 | 1 | 1 | 1 | 1 | 1 | 1 | 1 |
| wang hongyan2009 | 0 | 1 | 1 | 1 | 1 | 1 | 1 | 1 |
| tan jiehua2007 | 1 | 1 | 1 | 1 | 1 | 1 | 2 | 1 |
| zheng xiuxia2010 | 1 | 1 | 1 | 1 | 1 | 1 | 2 | 1 |
| zhang honghui 2008 | 1 | 1 | 1 | 1 | 1 | 1 | 1 | 1 |
| fan qinghua2011 | 1 | 1 | 1 | 1 | 1 | 1 | 2 | 1 |
| wang ying2010 | 0 | 1 | 1 | 1 | 1 | 1 | 1 | 1 |
| gao ying2009 | 0 | 1 | 1 | 1 | 1 | 1 | 1 | 1 |
| ma yong 2013 | 1 | 1 | 1 | 1 | 1 | 1 | 1 | 1 |
| kang meiyu2011 | 1 | 1 | 1 | 1 | 1 | 1 | 2 | 1 |
| meng xinling2014 | 1 | 1 | 1 | 1 | 1 | 1 | 2 | 1 |
| meng xinling2014 | 1 | 1 | 1 | 1 | 1 | 1 | 2 | 1 |
| lao meili2011 | 1 | 1 | 1 | 1 | 1 | 1 | 2 | 1 |
| sun hongxian2012 | 1 | 1 | 1 | 1 | 1 | 1 | 2 | 1 |
| cheng qi2013 | 1 | 1 | 1 | 1 | 1 | 1 | 1 | 1 |
| ji yong2015 | 1 | 1 | 1 | 1 | 1 | 1 | 1 | 1 |
| tang jiangping2014 | 1 | 1 | 1 | 1 | 1 | 1 | 2 | 1 |
| wei chongjuan2014 | 0 | 1 | 1 | 1 | 1 | 1 | 1 | 1 |
| ding ding 2014 | 1 | 1 | 1 | 1 | 1 | 1 | 2 | 1 |
| li haihong2015 | 1 | 1 | 1 | 1 | 1 | 1 | 2 | 1 |
| li chonghui2015 | 1 | 1 | 1 | 1 | 1 | 1 | 2 | 1 |
