# Supplementary figures and images for: Prevalence of dementia in the People’s Republic of China from 1985 to 2015: a systematic review and meta-regression analysis
Source: BMC Public Health. 2019 May 15;19:578. doi: 10.1186/s12889-019-6840-z (PMC6521412; doi:10.1186/s12889-019-6840-z)

The pooled prevalence of AD (A) /VAD (B) /dementia (C)

**A.**


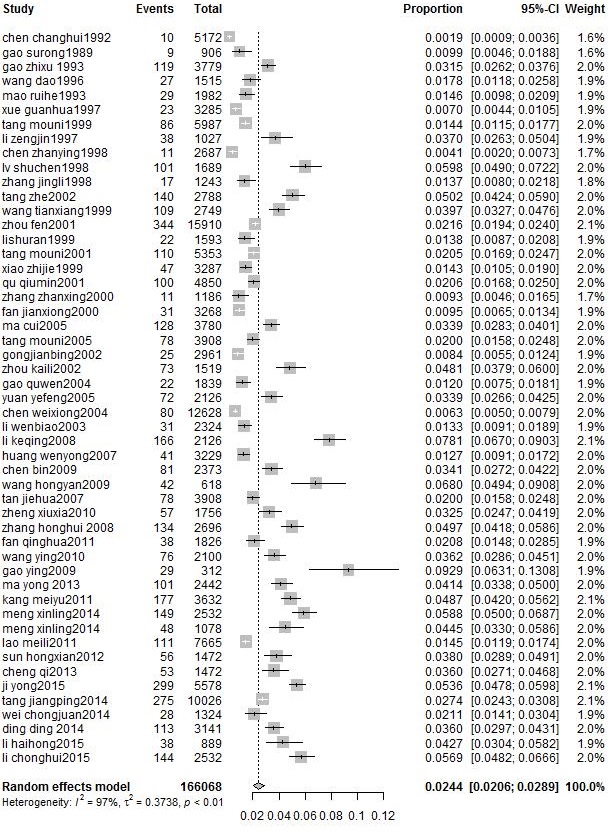


**B.**


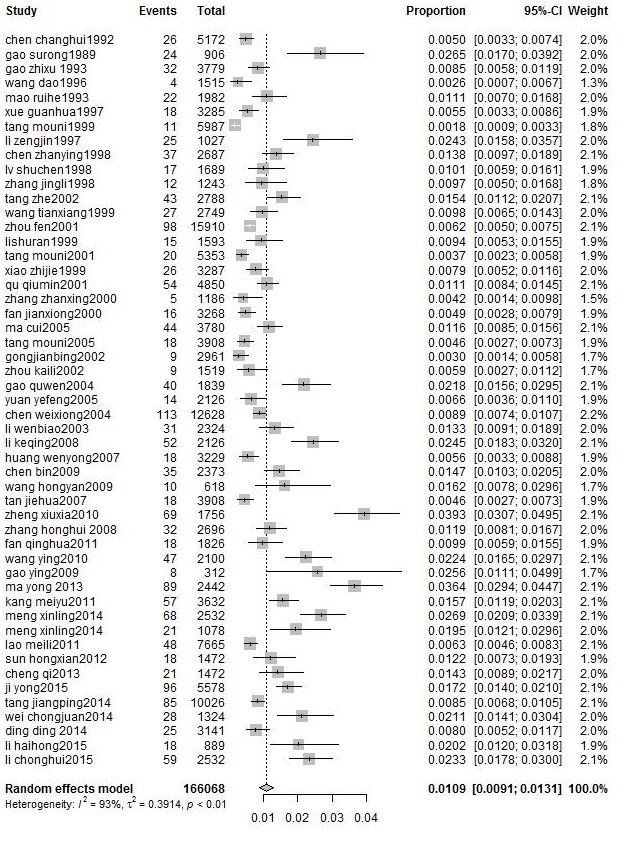


**C.**
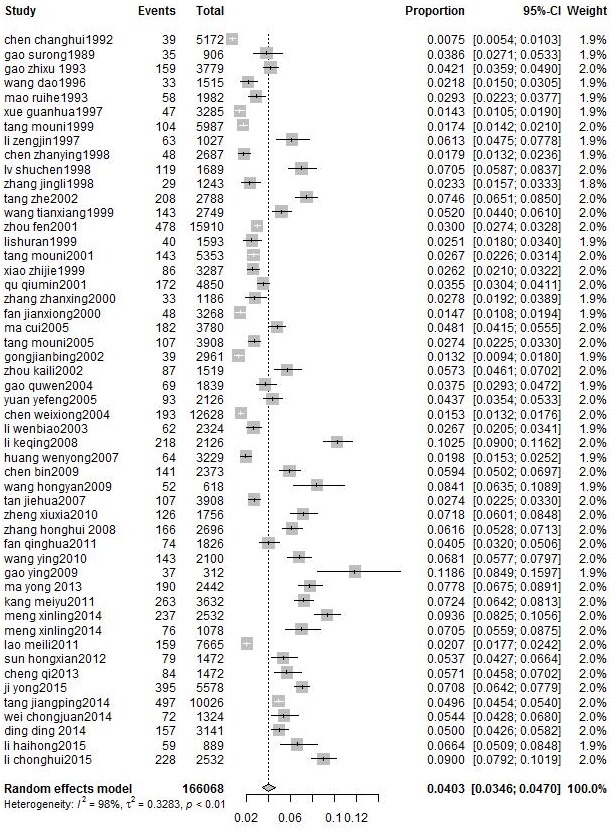

Supplement: Supplementary file 2 — The pooled prevalence of AD (A) /VAD (B) /dementia (C). (DOC 829 kb) [file 12889_2019_6840_MOESM2_ESM.doc]

The subgroup analysis the prevalence of AD (A) /VAD (B) /dementia (C) based on region

**A.**
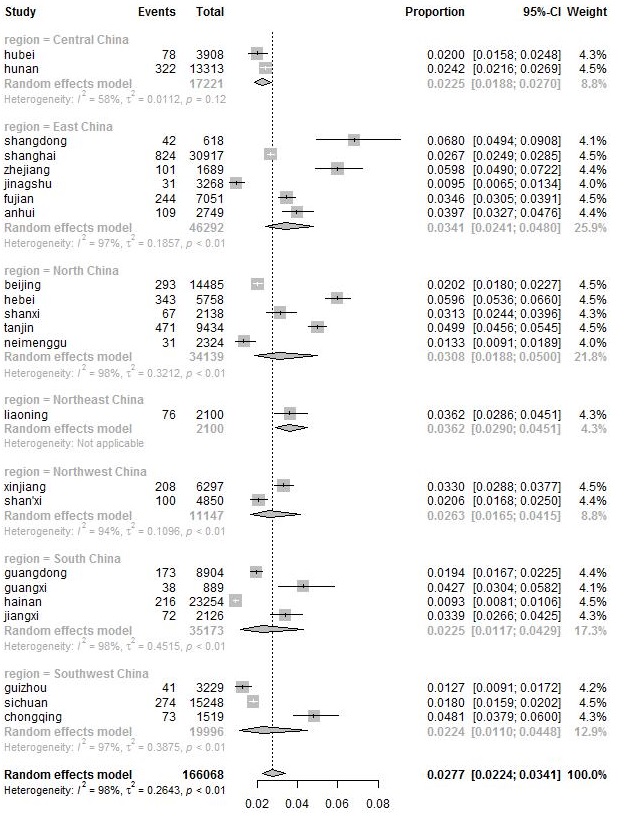


**B.**
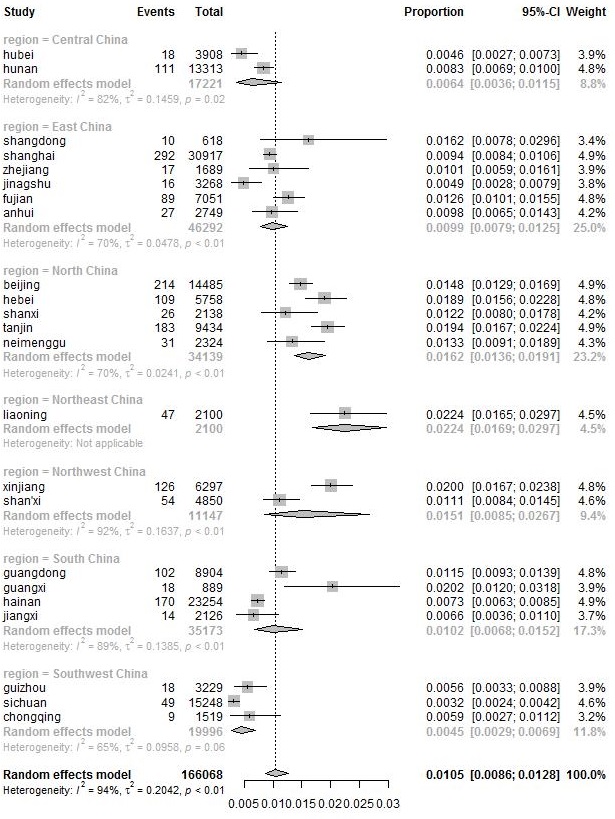


**C.**
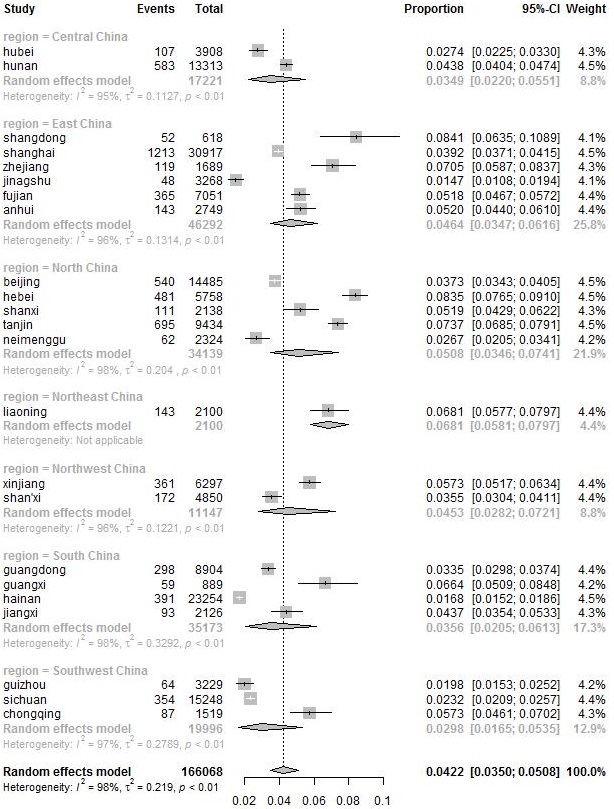

Supplement: Supplementary file 6 — The subgroup analysis the prevalence of AD (A) /VAD (B) /dementia (C) based on region. (DOC 543 kb) [file 12889_2019_6840_MOESM6_ESM.doc]

The subgroup analysis the prevalence of AD (A) /VAD (B) /dementia (C) based on published year

**A.
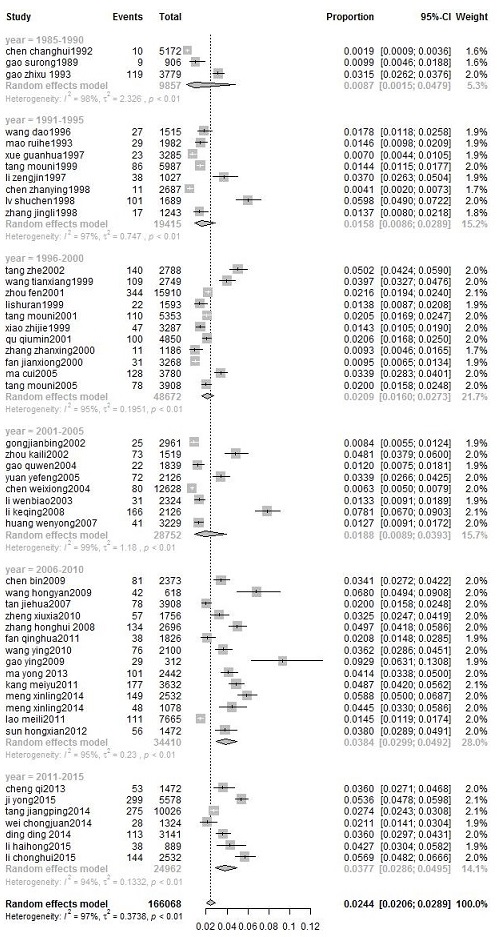
**

**B.**
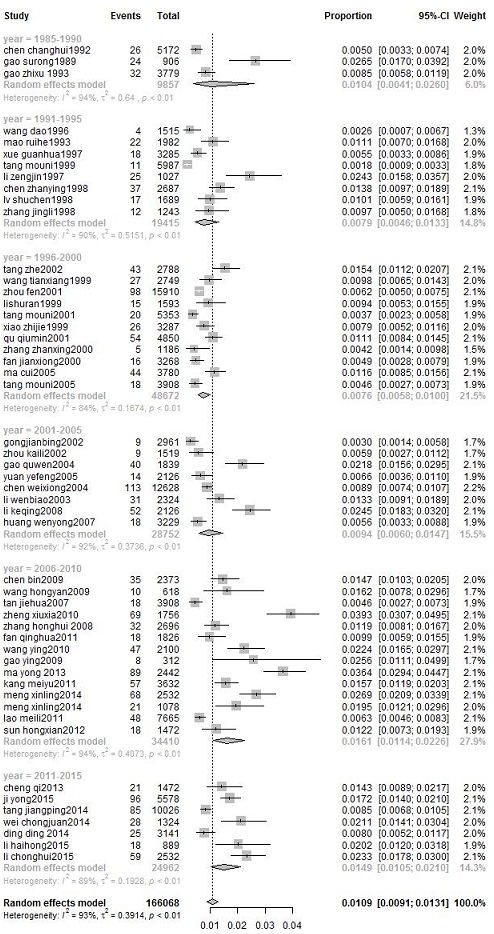


**C.
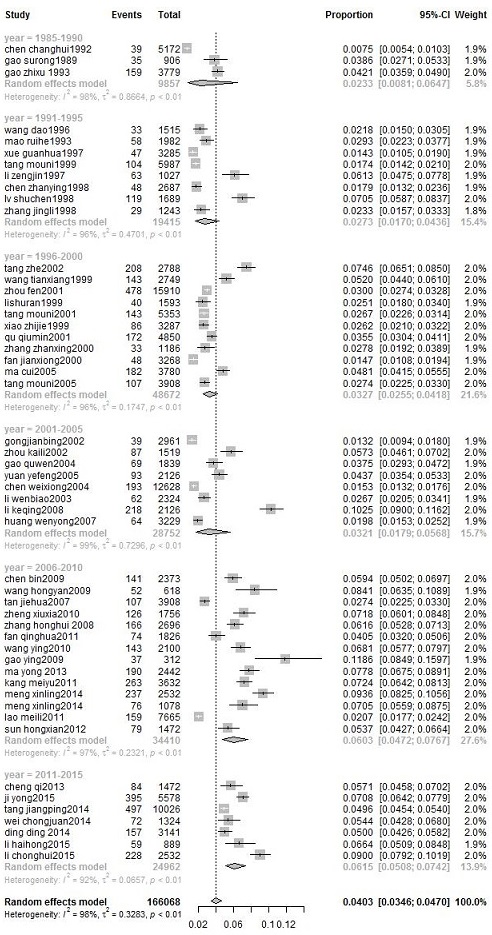
**

Supplement: Supplementary file 7 — The subgroup analysis the prevalence of AD (A) /VAD (B) /dementia (C) based on published year. (DOC 629 kb) [file 12889_2019_6840_MOESM7_ESM.doc]
