# Supplementary material for: Prevalence of dementia in the People’s Republic of China from 1985 to 2015: a systematic review and meta-regression analysis
Source: BMC Public Health. 2019 May 15;19:578. doi: 10.1186/s12889-019-6840-z (PMC6521412; doi:10.1186/s12889-019-6840-z)
Supplement: Supplementary file 3 — The prevalence of subtypes of dementia included in the studies. (DOC 108 kb) [file 12889_2019_6840_MOESM3_ESM.doc]

The prevalence of subtypes of dementia included in the studies

|  |  | | **AD** | **VD** | **Mixed** | **Parkinson** | **Hurt** | **Alcohol** | **Lewy bodies** | **Others** |
| --- | --- | --- | --- | --- | --- | --- | --- | --- | --- | --- |
| 1 | chen changhui1992 | | 10 | 26 | 2 |  |  |  |  | 1 |
| 2 | gao surong1989 | | 9 | 24 |  |  |  |  |  | 2 |
| 3 | gao zhixu 1993 | | 119 | 32 |  | 1 |  | 4 | 1 | 2 |
| 4 | wang dao1996 | | 27 | 4 |  |  |  |  |  | 2 |
| 5 | mao ruihe1993 | | 29 | 22 | 7 |  |  |  |  |  |
| 6 | xue guanhua1997 | | 23 | 18 |  |  | 1 |  |  | 5 |
| 7 | tang mouni1999 | | 86 | 11 | 3 |  | 1 | 2 |  | 1 |
| 8 | li zengjin1997 | | 38 | 25 |  |  |  |  |  |  |
| 9 | chen zhanying1998 | | 11 | 37 |  |  |  |  |  |  |
| 10 | lv shuchen1998 | | 101 | 17 |  |  |  |  |  | 1 |
| 11 | zhang jingli1998 | | 17 | 12 |  |  |  |  |  |  |
| 12 | tang zhe2002 | | 140 | 43 | 21 |  |  |  |  | 4 |
| 13 | wang tianxiang1999 | | 109 | 27 |  | 1 |  | 4 | 1 | 1 |
| 14 | zhou fen2001 | | 344 | 98 |  | 16 | 4 |  |  | 16 |
| 15 | Lishuran1999 | | 22 | 15 | 3 |  |  |  |  |  |
| 16 | tang mouni2001 | | 110 | 20 | 3 | 6 | 1 | 3 |  |  |
| 17 | xiao zhijie1999 | | 47 | 26 |  |  |  |  |  | 13 |
| 18 | qu qiumin2001 | | 100 | 54 |  | 4 | 1 | 1 |  | 12 |
| 19 | zhang zhanxing2000 | | 11 | 5 |  |  |  |  |  | 17 |
| 20 | Fan jianxiong2000 | | 31 | 16 |  |  |  |  |  | 1 |
| 21 | ma cui2005 | | 128 | 44 | 5 | 3 |  | 1 |  | 1 |
| 22 | tang mouni2005 | | 78 | 18 | 1 | 1 | 3 | 4 |  | 2 |
| 23 | gongjianbing2002 | | 25 | 9 | 5 |  |  |  |  |  |
| 24 | zhou kaili2002 | | 73 | 9 | 5 |  |  |  |  |  |
| 25 | gao quwen2004 | | 22 | 40 |  |  |  |  |  | 7 |
| 26 | yuan yefeng2005 | | 72 | 14 |  |  |  |  |  | 7 |
| 27 | chen xiongxiong2004 | | 80 | 113 |  |  |  |  |  |  |
| 28 | li wenbiao2003 | | 31 | 31 |  |  |  |  |  |  |
| 29 | li keqing2008 | | 166 | 52 |  |  |  |  |  |  |
| 30 | huang wenyong2007 | | 41 | 18 | 2 |  |  |  |  | 3 |
| 31 | chen bin2009 | | 81 | 35 | 10 |  |  |  |  | 15 |
| 32 | wang hongyan2009 | | 42 | 10 |  |  |  |  |  |  |
| 33 | tan jiehua2007 | | 78 | 18 | 1 | 1 | 3 | 4 |  | 2 |
| 34 | zheng xiuxia2010 | | 57 | 69 |  |  |  |  |  |  |
| 35 | zhang honghui 2008 | | 134 | 32 |  |  |  |  |  |  |
| 36 | fan qinghua2011 | | 38 | 18 |  |  |  |  |  | 18 |
| 37 | wang ying2010 | | 76 | 47 | 8 | 6 | 1 | 5 |  |  |
| 38 | gao ying2009 | | 29 | 8 |  |  |  |  |  |  |
| 39 | ma yong 2013 | | 101 | 89 |  |  |  |  |  |  |
| 40 | kang meiyu2011 | | 177 | 57 | 29 |  |  |  |  |  |
| 41 | meng xinling2014 | | 149 | 68 | 12 | 2 | 2 | 3 |  | 1 |
| 41 | meng xinling2014 | | 48 | 21 | 4 | 2 | 0 | 0 |  | 1 |
| 42 | lao meili2011 | | 111 | 48 |  |  |  |  |  |  |
| 43 | sun hongxian2012 | | 56 | 18 |  |  |  |  |  | 5 |
| 44 | cheng qi2013 | | 53 | 21 |  |  |  | 5 |  | 5 |
| 45 | ji yong2015 | | 299 | 96 |  |  |  |  |  |  |
| 46 | tang jiangping2014 | | 275 | 85 | 7 |  |  |  |  | 130 |
| 47 | wei chongjuan2014 | | 28 | 28 |  |  |  | 8 |  | 8 |
| 48 | ding ding 2014 | | 113 | 25 |  | 4 | 1 |  | 1 | 13 |
| 49 | li haihong2015 | | 38 | 18 | 3 |  |  |  |  |  |
| 50 | li chonghui2015 | | 144 | 59 | 22 |  |  |  |  | 3 |
|  | total subject | | 4227 | 1750 | 153 | 47 | 18 | 31 | 3 | 299 |
|  | Pooled prevalence | 0.0244 [0.0206;0.0289] | | 0.0109  [0.0091;0.0131] | 0.0023  [0.0019;0.0027] | 0.0009  [0.0007;0.0012] | 0.0004  [0.0002;0.0006] | 0.0008  [0.0005;0.0011] | 0.0003  [0.0001;0.0009] | 0.0029  [0.0026;0.0033] |
