# Supplementary material for: Prevalence of dementia in the People’s Republic of China from 1985 to 2015: a systematic review and meta-regression analysis
Source: BMC Public Health. 2019 May 15;19:578. doi: 10.1186/s12889-019-6840-z (PMC6521412; doi:10.1186/s12889-019-6840-z)
Supplement: Supplementary file 4 — The prevalence of dementia in terms of age in China from 1985 to 2015. (DOC 39 kb) [file 12889_2019_6840_MOESM4_ESM.doc]

The prevalence of dementia in terms of age in China from 1985 to 2015

|  | Alzheimer's Disease | | | Vascular Dementia | | | Dementia | | |
| --- | --- | --- | --- | --- | --- | --- | --- | --- | --- |
|  | male | female | total | male | female | total | male | female | total |
| 55-59 | 0.0000[0.0000; 0.0018] | 0.0000[0.0000; 0.0029] | 0.0000[0.0000; 0.0011] | 0.0026[0.0005; 0.0075] | 0.0010[0.0000; 0.0057] | 0.0019[0.0005; 0.0048] | 0.0011[0.0002; 0.0032] | 0.0036[0.0019; 0.0062] | 0.0025[0.0015; 0.0041] |
| 60-64 | 0.0066 [0.0024; 0.0177] | 0.0092 [0.0040; 0.0209] | 0.0055 [0.0036; 0.0083] | 0.0125 [0.0028; 0.0536] | 0.0092 [0.0040; 0.0209] | 0.0055 [0.0037; 0.0084] | 0.0095 [0.0025; 0.0350] | 0.0121 [0.0035; 0.0414] | 0.0091 [0.0055; 0.0149] |
| 65-69 | 0.0053 [0.0018; 0.0152] | 0.0112 [0.0059; 0.0212] | 0.0085 [0.0060; 0.0121] | 0.0081 [0.0036; 0.0177] | 0.0058 [0.0023; 0.0145] | 0.0073 [0.0052; 0.0102] | 0.0142 [0.0044; 0.0451] | 0.0177 [0.0079; 0.0389] | 0.0168 [0.0123; 0.0229] |
| 70-74 | 0.0166 [0.0103; 0.0267] | 0.0257 [0.0183; 0.0359] | 0.0208 [0.0161; 0.0268] | 0.0156 [0.0108; 0.0226] | 0.0141 [0.0072; 0.0274] | 0.0116 [0.0086; 0.0156] | 0.0359 [0.0206; 0.0618] | 0.0392 [0.0240; 0.0635] | 0.0337 [0.0277; 0.0408] |
| 75-79 | 0.0316 [0.0263; 0.0379] | 0.0486 [0.0425; 0.0554] | 0.0421 [0.0346; 0.0511] | 0.0146 [0.0071; 0.0297] | 0.0224 [0.0160; 0.0313] | 0.0177 [0.0132; 0.0235] | 0.0562 [0.0385; 0.0815] | 0.0818 [0.0590; 0.1124] | 0.0643 [0.0559; 0.0739] |
| 80-84 | 0.0636 [0.0538; 0.0752] | 0.1111 [0.0933; 0.1318] | 0.0993 [0.0816; 0.1202] | 0.0382 [0.0267; 0.0546] | 0.0329 [0.0197; 0.0545] | 0.0323 [0.0230; 0.0452] | 0.0997 [0.0732; 0.1344] | 0.1338 [0.1150; 0.1553] | 0.1295 [0.1070; 0.1560] |
| 85-89 | 0.1262 [0.105; 0.151] | 0.1746 [0.1381; 0.2184] | 0.1612 [0.1328; 0.1944] | 0.0593 [0.0341; 0.1010] | 0.0399 [0.0175; 0.0885] | 0.0366 [0.0178; 0.0737] | 0.1756 [0.1495; 0.2052] | 0.2248 [0.1882; 0.2661] | 0.2177 [0.1919; 0.2460] |
| 90-94 | 0.1558 [0.1094; 0.217] | 0.3182 [0.2761; 0.3636] | 0.2594 [0.2035; 0.3244] | 0.1237 [0.0624; 0.2303] | 0.0452 [0.0126; 0.149] | 0.0585 [0.0195; 0.1627] | 0.2289 [0.1734; 0.2959] | 0.3618 [0.3177; 0.4083] | 0.3822 [0.3291; 0.4382] |
| ≥ 95 | 0.6162 [0.2363; 0.8928] | 0.5237 [0.3181; 0.7216] | 0.3958 [0.2220; 0.6005] | 0.1285 [0.0176; 0.5475] | 0.0634 [0.0128; 0.2613] | 0.1237 [0.0198; 0.4959] | 0.6162 [0.2363; 0.8928] | 0.5237 [0.3181; 0.7216] | 0.5169 [0.3368; 0.6926] |
