# Supplementary material for: Prevalence of dementia in the People’s Republic of China from 1985 to 2015: a systematic review and meta-regression analysis
Source: BMC Public Health. 2019 May 15;19:578. doi: 10.1186/s12889-019-6840-z (PMC6521412; doi:10.1186/s12889-019-6840-z)
Supplement: Supplementary file 5 — Prevalence of dementia due to sex, education, occupation and residence difference. (DOC 36 kb) [file 12889_2019_6840_MOESM5_ESM.doc]

Prevalence of dementia due to sex, education, occupation and residence difference

|  | **Alzheimer’s disease** | **Vascular dementia** | **Total dementia** |
| --- | --- | --- | --- |
| **Sex** |  |  |  |
| **Male** | 0.0158 [0.0146; 0.0170] | 0.0137 [0.0126; 0.0148] | 0.0305[0.0293; 0.0317] |
| **Female** | 0.0324 [0.0308; 0.0341] | 0.0100 [0.0091; 0.0110] | 0.0455[0.0441; 0.0469] |
| **Education** |  |  |  |
| **Illiterate** | 0.0606 [0.0408; 0.0891] | 0.0242 [0.0157; 0.0371] | 0.0591 [0.0443; 0.0783] |
| **Primary school** | 0.0221 [0.0139; 0.0350] | 0.0144 [0.0102; 0.0204] | 0.0572 [0.0425; 0.0765] |
| **Junior high school** | 0.0155 [0.0059; 0.0404] | 0.0112 [0.0025; 0.0494] | 0.0492 [0.0292; 0.0818] |
| **Senior high school** | 0.0144 [0.0083; 0.0247] | 0.0108 [0.0052; 0.0221] | 0.0310 [0.0113; 0.0825] |
| **College** | 0.0068 [0.0031; 0.0148] | 0.0067 [0.0034; 0.0132] | 0.0300 [0.0183; 0.0489] |
| **Residence** |  |  |  |
| **city** | 0.0255 [0.0186; 0.0348] | 0.0125 [0.0083; 0.0187] | 0.0411 [0.0327; 0.0514] |
| **Rural** | 0.0265 [0.0180; 0.0389] | 0.0115 [0.0079; 0.0167] | 0.0487 [0.0380; 0.0623] |
| **Occupation** |  |  |  |
| **Worker** | 0.0104 [0.0076; 0.0142] | 0.0109 [0.0079; 0.0150] | 0.0387 [0.0233; 0.0634] |
| **Farmer** | 0.0143 [0.0070; 0.0289] | 0.0131 [0.0066; 0.0259] | 0.0417 [0.0301; 0.0577] |
| **Officer** | 0.0222 [0.0182; 0.0270] | 0.0128 [0.0097; 0.0169] | 0.0298 [0.0145; 0.0602] |
| **Housewife** | 0.0475 [0.0355; 0.0632] | 0.0141 [0.0082; 0.0242] | 0.0740 [0.0517; 0.1049] |
|  |  |  |  |
|  |  |  |  |
