# Supplementary material for: Prevalence of dementia in the People’s Republic of China from 1985 to 2015: a systematic review and meta-regression analysis
Source: BMC Public Health. 2019 May 15;19:578. doi: 10.1186/s12889-019-6840-z (PMC6521412; doi:10.1186/s12889-019-6840-z)
Supplement: Supplementary file 8 — Univariate meta-regression of the prevalence of dementia/AD/VAD. (DOC 40 kb) [file 12889_2019_6840_MOESM8_ESM.doc]

Univariate meta-regression of the prevalence of dementia/AD/VAD

| **Dementia** | **Metaregression Coefficient (%)** | **95%CI** | **P** |
| --- | --- | --- | --- |
| **Dementia** |  |  |  |
| **Year of publication**  **(year<2000 vs. year>=2000)** | 0.5808 | 0.2738 - 0.8878 | 0.0002 |
| **Geographical region**  **(north vs. south)** | -0.2904 | -0.5886 - 0.0078 | 0.0563 |
| **Living area**  **(city vs. rural)** | 0.0809 | -0.6346 - 0.7963 | 0.8247 |
| **Living area**  **(city vs. city+rural)** | -0.1301 | -0.4726 - 0.2125 | 0.4567 |
| **Sample size**  **(<3000 vs. >=3000)** | 0.6028 | 0.3079 - 0.8977 | <.0001 |
| **Diagnostic method**  **(DSM-III/IV/IV-R vs. others)** | -0.3791 | -0.8737 - 0.1155 | 0.1331 |
| **AD** |  |  |  |
| **Year of publication**  **(year<2000 vs year>=2000)** | 0.6596 | 0.3151-1.0041 | 0.0002 |
| **Geographical region**  **(north vs. south)** | -0.2597 | -0.5813-0.0618 | 0.1134 |
| **Living area**  **(city vs rural)** | 0.3665 | -0.3088-1.0417 | 0.2874 |
| **Living area**  **(city vs. city+rural)** | -0.0438 | -0.4126-0.3250 | 0.8160 |
| **Sample size**  **(<3000 vs. >=3000)** | 0.3734 | 0.0326-0.7143 | 0.0318 |
| **Diagnostic method**  **(DSM-III/IV/IV-R vs others)** | -0.2350 | -0.8118-0.3418 | 0.4246 |
| **VAD** |  |  |  |
| **Year of publication**  **(year<2000 vs. year>=2000)** | 0.4886 | 0.1389-0.8384 | 0.0062 |
| **Geographical region**  **(north vs south)** | -0.6978 | -1.0217--0.3739 | <.0001 |
| **Living area**  **(city vs. rural)** | -0.4094 | -1.1191-0.3003 | 0.2582 |
| **Living area**  **(city vs city+rural)** | -0.3823 | -0.7656-0.0011 | 0.0506 |
| **Sample size**  **(<3000 vs. >=3000)** | 0.4933 | 0.1735- 0.8130 | 0.0025 |
| **Diagnostic method**  **(DSM-III/IV/IV-R vs. others)** | 0.0892 | -0.5240- 0.7023 | 0.7756 |
